# Soil Erosion Thickness and Seasonal Variations Together Drive Soil Nitrogen Dynamics at the Early Stage of Vegetation Restoration in the Dry-Hot Valley

**DOI:** 10.3390/microorganisms12081546

**Published:** 2024-07-28

**Authors:** Wenxu Liu, Zhe Chen, Li Rong, Xingwu Duan, Yuhong Qin, Zhenjie Chun, Xuening Liu, Jia Wu, Zihao Wang, Taicong Liu

**Affiliations:** 1 Yunnan Key Laboratory of Soil Erosion Prevention and Green Development, Yunnan University, Kunming 650500, China; luckyxxxu@163.com; 2 Yuanjiang Dry-Hot Valley Water and Soil Conservation Observation and Research Station of Yunnan Province, Institute of International Rivers and Eco–Security, Yunnan University, Kunming 650500, China; zhechen2019@ynu.edu.cn (Z.C.); qyh1998794@163.com (Y.Q.); czj4242@163.com (Z.C.); lxnsherryl@163.com (X.L.); wu_jia0908@163.com (J.W.); taicongliu@163.com (T.L.); 3 School of Ecology and Environmental Science, Yunnan University, Kunming 650500, China; 4 School of Earth Science, Yunnan University, Kunming 650500, China; 17332699453@163.com

**Keywords:** arbuscular mycorrhizal fungi, diazotrophs, nitrogen, soil erosion thickness

## Abstract

By changing the physicochemical and biological properties of soil, erosion profoundly affects soil nitrogen levels, but knowledge about the erosion impact on soil nitrogen (N) dynamics is still rather incomplete. We compared soil N contents at the early stage of vegetation self-restoration in response to soil erosion thickness (0, 10, 20, 30 and 40 cm), by conducting a simulated erosion experiment on sloping arable land in the dry-hot valley of Yunnan Province, southwestern China. The results showed total nitrogen (TN), ammonium nitrogen (NH_4_^+^-N) and nitrate nitrogen (NO_3_^−^-N) contents reduced with increasing soil erosion thickness and decreased significantly at the soil erosion thickness of 10, 40 and 10 cm in the rainy season and 30, 10 and 10 cm in the dry season compared with 0 cm. Structural equation modeling (SEM) indicated that soil erosion thickness and seasonal variation were the important drivers of mineral nitrogen (NH_4_^+^-N and NO_3_^−^-N) content. Soil erosion thickness indirectly affected mineral nitrogen through negative on TN, carbon content and Diazotrophs (*nifH* genes). Dry–wet season change had an effect on mineral nitrogen mediated by arbuscular mycorrhizal fungi (AMF) and *nifH* genes. We also found AMF had a promotion to *nifH* genes in eroded soil, which can be expected to benefit nitrogen fixing. Our findings highlight the importance of considering soil erosion thickness and sampling time for nitrogen dynamics, in particular, the investigation of nitrogen limitation, in the early stage of vegetation self-restoration.

## 1. Introduction

Soil erosion is a widespread, global phenomenon, and accelerating erosion is responsible for about half of all soil degradation worldwide [1]. The annual amount of topsoil being lost due to soil erosion may now exceed 75 billion tons globally [2], which could trigger considerable nutrient losses and exacerbate the nutrient limitations to plant growth [3]. In particular, soil erosion can cause soil nitrogen (N) losses of as high as 160 kg ha^−1^ yr^−1^ [4]. Many recent studies have emphasized soil erosion’s role in nitrogen cycles, leading to a synthesis of how it exerts important control on nitrogen dynamics [5,6,7]. The three-step process of soil erosion consisting of detachment, transport and deposition can affect the availability, stock, nature and persistence of soil nitrogen [8]. Most part studies have explicitly accounted for the influence of soil erosion on nitrogen loss in runoff plots [9,10] or on N mineralization in a micro-plot through artificial rainfall simulation experiments [11] as well as erosion and deposition on the redistribution of nitrogen fractions in landscapes [12,13]. It is noted that with the increase in soil erosion thickness, both the soil layer depth and soil nutrients contents decrease [14], along with soil aggregates and soil microorganisms [15,16]. Subsequently, the changes in soil physicochemical and biological properties, resulting from the degree of soil erosion, will inevitably impact soil N dynamics. Although many studies have focused on the soil erosion on N loss and transformation, the effects of soil erosion thickness on soil N dynamics have been neglected. Since soil N is one of the essential elements for plant nutrition, it can act as a limiting factor for the growth of many plant species, such that soil N supply capacity largely determines the course and success of ecological restoration [17]. Hence, N resident in eroded soil is crucial for plant fitness [18], and it is imperative to gain a sound understanding the effect of soil erosion thickness on soil N dynamics.

Most (>99%) soil nitrogen is contained in dead organic matter derived from plants, animals and microbes. In natural ecosystems, most nitrogen absorbed by plants becomes available through the decomposition of organic matter. More than 90% of the nitrogen macromolecules in soil exist in the form of organic nitrogen [19]. Some studies have shown that some plants can utilize small insoluble organic nitrogen (DON) [20,21], but many plants tend to absorb and utilize mineral nitrogen (ammonium nitrogen and nitrate nitrogen) [22,23]. Microorganisms can help optimize resource availability. It is well known that ammonium nitrogen (NH_4_^+^-N) and nitrate nitrogen (NO_3_^−^-N) are primarily regulated by microbe-mediated mineralization, immobilization, nitrification and denitrification [24]. It has been well documented that soil N dynamics are closely related to litter quality, phsicochemical soil properties and microbial activity, and at large climatic gradients, climatic factors can affect both the substrate availability and oxygen in the soil, thus influencing soil N dynamics [24]. Although the effects of an individual factor on soil N dynamics are relatively well studied, the complex interaction among these factors and soil erosion in regulating N dynamics remains unclear, hindering our ability to understand the limits of vegetation restoration.

The dry-hot valleys are type of a unique geographical region found in southwestern China [25]. These are tropical and subtropical deep river valleys, mainly distributed in the watersheds of the Jinsha River, Yuanjiang River, Nujiang River and their tributaries in Yunnan Province. Among them, the dry-hot valley area in the Yuanjiang River’s catchment is the most concentrated and contiguously distributed in Yunnan Province [26]. This region has a steep topography, and a substantial proportion of its land is under cultivation on steep slopes. Its extensive steep terrain and concentrated rainfall combined with steep slope cultivation render the region vulnerable to severe erosion, whose average soil erosion modulus is 12.56 t ha^−1^ a^−1^ [27]. Consequently, nutrient deficits are common in dry-hot valleys, with greater soil erosion leading to a greater deficit of nutrients for plants [28]. To mitigate and control soil erosion, afforestation has been carried out in many zones of the dry-hot valley over the last 50 years, and impressive outcomes have been achieved [29,30]; however, on some seriously degraded sloping land, afforestation has been proved to be ineffective [31]. To understand how soil erosion thickness affects N dynamics under vegetation restoration, we established field plots that simulated different erosion thickness on sloping arable land in a typical dry-hot valley, where vegetation succession has been allowed proceed naturally, and we presented a persistent monitoring to elucidate the effects of soil erosion thickness on N dynamics. Our objectives were (1) to explore the variation in TN, NH_4_^+^-N and NO_3_^−^-N dynamics in response to soil erosion thickness and (2) to determine whether soil erosion thickness drives N dynamics.

## 2. Materials and Methods

### 2.1. Study Site

The plots of simulated soil erosion thickness were established in an abandoned corn-field at Yuanjiang Dry-Hot Valley Water and Soil Conservation Observation and Research Station of Yunnan Province (23°58′5″ N, 101°38′55″ E) in southwestern China. This station is located in a typical dry-hot valley of the Yuanjiang–Red River Basin. This region has an average elevation of 542 m and slope of 27° and a typical plateau monsoon climate, with a multi-year average temperature of 23.9 °C, precipitation of 781 mm and evaporation of 2892 mm. From October 2020 to September 2021, the total precipitation was 651.30 mm, and the average temperature was 24.64 °C (Figure S1). As such, the region is extremely arid and has two distinct seasons, namely, the dry and rainy season [32]. The latter (late May to mid-October) receives more than 80% of yearly rainfall, whereas the dry season lasts from late October to mid-May of the following year [33]. Soils of the area are classified as Ferralosols in Chinese Soil Taxonomy [34] and Ultisols in United States Soil Taxonomy [35]. The vegetation types in this study area are mainly shrubs and grass bushes. Based on our vegetation survey of simulated erosion thickness plots, the dominant species are *Bidens pilosa*, *Cajanus scarabaeoides*, *Heteropogon contortus* and *Taraxacum mongolicum*.

### 2.2. Establishment of the Simulated Erosion Thickness Plots

**Figure 1:**
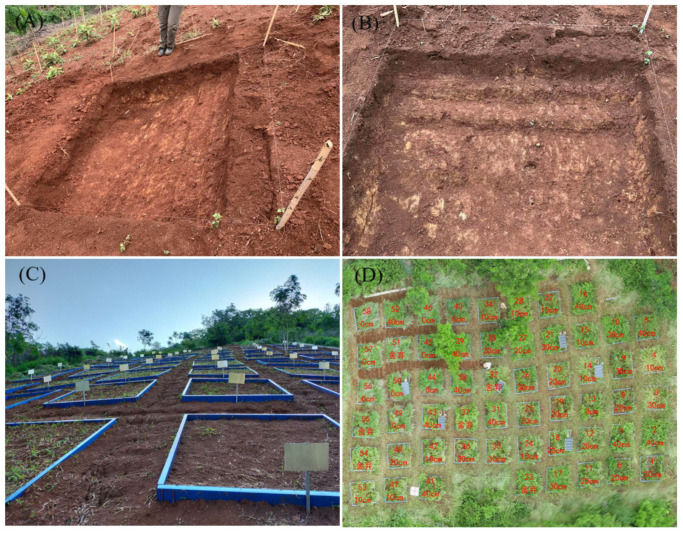
Images of the simulated erosion plots with differing erosion thickness. Steps in the construction process of simulated erosion plots (**A**,**B**); Side and overhead views of the 50 established plots (**C**,**D**); 10 plots per erosion thickness level.

The pre-field survey results indicated that the effective soil layer thickness of sloping arable land in the dry-hot valley was mostly around 40 cm. In April 2020, the method of “Cutting-and-Filling” was used to establish artificial simulated erosion plots. Each plot was 2 × 2 m in size, and all had the same soil, slope, aspect, elevation and geomorphic location. According to the soil profile characteristics of sloping arable land without erosion, the levels of erosion thickness were set to 0, 10, 20, 30 and 40 cm, these corresponding to soil erosion intensity of no erosion (control), light erosion, moderate erosion, strong erosion and severe erosion, respectively. Each treatment was replicated 10 times, for a total of 50 simulated plots (Figure 1), where the treatments were randomly assigned [15,36,37].

#### 2.2.1. Principles of the Construction and Simulation Process of the Erosion Plots

In cultivated land, surface soil in the cultivated horizon is easily lost through erosion, while the plow pan soil is usually plowed and mixed into the original cultivated horizon. As a result, the thickness of the cultivated horizon may remain constant, but its composition continues to change. Thus, the impact of tillage on soil layers in cultivated land should be considered when simulating erosion thicknesses. The original soil profile was defined as $h_{0}$, $h_{1}$, $h_{2}$, $h_{3}$, …, and $h_{i}$ layers. $h_{0}$ represented the original cultivation horizon, defined at 20 cm. We hypothesized that the annual soil erosion thickness was equal to the thickness of soil from the plow pan to the cultivation horizon and that the annual soil erosion thickness had no interannual variation. After *n* years of erosion, the thickness of the original *i* soil layer remaining in the cultivated horizon can be calculated using the formula of Wang et al. (2009) and Hou et al. (2014) [36,37].

(1) $$h_{i}^{\prime }=h_{i}\times \left(1\right.-\frac{d}{m}{)}^{n}$$

where $h_{i}^{\prime }$ is the thickness of the original *i* soil layer remaining in the cultivated horizon (cm) which has been plowed and eroded for *n* years; $h_{i}$ is the soil thickness of the original *i* layer (cm), *i* = 0, 1, 2, 3… *n*; *d* is the average annual erosion thickness, defined as 0.1 cm a^−1^ for Yuanjiang River Basin based on the report of the First National Census for Water in China; and *m* is the thickness of the cultivated horizon (cm), defined as 20 cm. The simulated erosion d thicknesses are 0 (control), 10, 20, 30 and 40 cm. The thickness of the soil layer in the original soil profile below $h_{0}$ was defined as 10 cm for $h_{0}$, $h_{1}$, $h_{2}$, $h_{3}$, …, and $h_{i}$ layer. Thus, the soil components of the cultivated horizon (cm) for simulated erosion thicknesses (10–40 cm) were calculated using Equation (1).

After *n* years of erosion, the components of the new cultivated horizon at different simulated erosion thicknesses were calculated with Equation (2).

(2) $$h=h_{0}\times {\left(1-\frac{d}{m}\right)}^{n}+\ldots +{\;h}_{i-1}\;\times {\left(1-\frac{d}{m}\right)}^{n-j}+{\;h}_{i}^{\prime }$$

where *h* is 20 cm, the thickness of the new cultivated horizon (cm), *j* is the erosion time (in years) when the $h_{i-1}$ soil layer was ploughed and eroded, and *d* and *m* are as above. The thickness of the original soil layer remaining in the new cultivated horizon for different simulated erosion thicknesses are given in Supplementary Material Table S2. For example, for a simulated soil loss of 10 cm, the remaining thickness of the original cultivated horizon $h_{0}$ in the new cultivated horizon was computed as 12.12 cm, with the new cultivated layer now extending 7.88 cm into the original 20–30 cm layer. Similarly, the inferred thickness of the original cultivated horizon after 20 cm of soil loss and continued annual mixing of topsoil and subsoil material by tillage, would be 7.34 cm. The corresponding value for an erosion thickness of 40 cm would be 2.69 cm (Table S2). Although the components of the cultivated horizon at different erosion thicknesses can be calculated with Equation (2), it is very difficult to accurately cut a thin layer of soil for collection and mixing, because many of the remaining thicknesses in cultivated horizons are only a few centimeters. Any layer of soil has the characteristics of length, width and height. The volume of the original soil layer remaining in the new cultivated layer was *(*$h_{i}^{\prime }\;$*/* 100*)* × *a* × *b* (m^3^), where *a* and *b* are the length and width of simulated erosion plots (this simulated erosion plot was 2 m length and 2 m width). Thus, the thickness of the original soil layer remained in the new cultivated layer can be converted into the volume composition of the original soil layer (Table S3). Then, $h_{i}^{\prime }$ was set to represent thickness ($h_{i}$) of the original soil layer, and the length and width were $a^{\prime }$ and $b^{\prime }$. Using this information, we projected a solid block with a volume of $(h_{i}/100)\times a^{\prime }\times b^{\prime }$ *(*m^3^*)* and then set it equal to *(*$h_{i}^{\prime }\;$*/*100*)* × *a* × *b (*m^3^*)*. We know the height and volume of the soil as $(h_{i}/100)\times a^{\prime }\times b^{\prime }$; if any value of $a^{\prime }$ or $b^{\prime }$ is given (with a fixed length of 2 m in this experiment), the value of the other one can be calculated. In this way, the problem of cutting a thin layer of soil for collection was transformed into a problem of cutting soil with constant thickness, length of 2 m and width of *b*′, which was very convenient to handle (Table S4). 

#### 2.2.2. Parent Materials Added

Because different thickness of soil were removed, it would inevitably lead to differences in the height of each plot from the original horizontal ground. This would not only cause different conditions among different erosion plots, such as temperature and moisture, but also lead to water accumulation in the rainy season in some erosion plots with large erosion thicknesses. Considering that the original soil profile below 40 cm was parent material, proportionate amounts of subsoil materials were added above 40 cm in simulated erosion plots to match each loss of topsoil and ensure that the surface height of each erosion plot was at the same level. For example, when the erosion thickness was 10 cm, soil parent material with a thickness of 10 cm should be added above the original profile of 40 cm. The construction process and established plots are shown in Figure 1. After the establishment of simulated erosion plots, vegetation was allowed to regrow naturally through natural succession.

### 2.3. Soil and Plant Samples, Collection and Measurement

Soil samples were collected from within the simulated erosion plots on 8 October 2020 and 8 April 2021, respectively. Five soil cores were taken from the upper soil layer (0–20 cm) in each plot using a soil auger (5 cm diameter) and mixed into one composite soil sample after removing any visible debris and stones. Thus, a total of 50 soil samples were collected and taken to the laboratory. There, a portion of each composite sample was stored in a refrigerator at 4 °C for the determination of NH_4_^+^-N, NO_3_^−^-N and dissolved organic carbon (DOC); the other portion was placed in a 2 mL centrifuge tube and stored in a freezer at −80 °C for its later molecular analysis. The leftover portions of the composite samples were then passed through 2.0 and 0.149 mm sieves after air-drying them, for the subsequent determination of their soil physical and chemical properties. One quadrat of 0.5 × 0.5 m per plot were sampled for the determination of the aboveground biomass. The quadrat was chosen randomly, and all aboveground biomass within the quadrant was cut at soil level. A total of 50 samples were collected in the vellum bags.

Soil particle size distribution, namely, for sand (2–0.02 mm), silt (0.02–0.002 mm) and clay (<0.002 mm) [38,39], was measured by applying the hydrometer method [40]. Soil pH was measured in a 1:2.5 soil/water suspension with the pH meter (METTLER-S220, Labcan Scientific Supplies Co., Ltd., Shanghai, China). Soil moisture (SM) was determined by oven-drying the soil at 105 °C to a constant weight. Soil organic carbon (SOC) was measured by converting total organic carbon into CO_2_ via high-temperature combustion and catalytic oxidation with an organic carbon analyzer (VarioTOC, Elementar, Langenselbold, Germany). TN was determined using the Kjeldahl method, while total phosphorus (TP) and total potassium (TK) were determined using the molybdenum antimony colorimetric and flame photometer methods, respectively; available phosphorus (AP) was quantified using a spectrophotometer; available potassium (AK) was quantified using a flame photometer after preparing the samples using an ammonium acetate solution [41]; finally, DOC, NH_4_^+^-N and NO_3_^−^-N were measured on a continuous-flow Auto Analyzer (AA3, Bran-Luebbe, Nordersteld, Germany). The plant samples were placed in a constant-temperature drying oven at 105 °C for 30 min, dried at 65 °C to constant weight and then reweighed to calculate the aboveground biomass.

### 2.4. Soil Microbial DNA Extraction, PCR Amplification and Sequencing

The total DNA was extracted from 0.25 g soil samples, by using the E.Z.N.A.^®^ soil DNA Kit (Omega Bio-tek, Norcross, GA, USA) according to the manufacturer’s instructions. The extracted DNA was evaluated on a 1% agarose gel. In addition, the quality and quantity of DNA extracts (final volume, 100 μL) were checked using a NanoDrop 2000 spectrophotometer (Thermo Scientific, Wilmington, NC, USA). To generate the libraries of Arbuscular mycorrhizal fungi (AMF) and Diazotrophs (*nifH* genes), their PCR amplifications were, respectively, carried out using the primer pairs AMV4-5NF (5′-AAGCTCGTAGTTGAATTTCG-3′)/AMDGR (5′-CCCAACTATCCCTATTAATCAT-3′) [41,42] and nifHF (5′-AAAGGYGGWATCGGYAARTCCACCAC-3′)/nifHR (5′-TTGTTSGCSGCRTACATSGCCATCAT-3′) [43]. The ensuing PCR products were separated and purified by gel electrophoresis (2% agarose in 1 × TAE), by using the AxyPrep DNA Gel Extraction Kit (Axygen Biosciences, Union City, CA, USA), and then quantified with a Quantus™ Fluorometer (Promega, Madison, WI, USA). Purified amplicons were pooled in equimolar quantities and sequencing libraries were assembled using the NEXTFLEX Rapid DNA-Seq Kit for Illumina (Illumina Inc., San Diego, CA, USA), by following the manufacturer’s recommendations. Paired-end sequencing was performed on an Illumina MiSeq PE300 sequencing platform (Illumina, San Diego, CA, USA), according to standard protocols, by the Majorbio Bio-Pharm Technology Co., Ltd. (Shanghai, China).

### 2.5. Statistical Analysis

Shapiro–Wilk (S–W) and homogeneity tests were used to check the data for their normal distribution and homogeneity of variance. If the variance was homogeneous, a one-way analysis of variance (ANOVA), followed by Fisher’s least significant difference (LSD) test, was applied to determine differences in soil physicochemical properties, mineral nitrogen, aboveground biomass and microbial alpha diversity among the five erosion thickness treatments. These analyses were implemented in SPSS 24.0 for Windows (SPSS Inc., Chicago, IL, USA). The beta diversity analysis was based on unweighted UniFrac data [44]. Nonmetric multidimensional scaling (NMDS) was used to infer patterns in microbial community composition within and among the five erosion thickness treatments, using Bray–Curtis distances at the genus level, with the “vegan” package for R software (v4.1.1; http://cran.r-project.org/, accessed on 15 October 2022). Using this package as well, PERMANOVA (permutation multivariate analysis of variance), based on 999 permutations, was used with Bray–Curtis distances and the Adonis function to test for significant differences in soil microbial community composition among different erosion thickness treatments.

To explore the complex relationships between soil thickness, soil properties, plant characteristics and nitrogen dynamics, we built a structural equation model (SEM). The partial-least-squares (PLS) method was used for the SEM’s path analysis (PLS-PA). To estimate the accuracy of PLS parameter estimates, nonparametric bootstrapping was performed, with 95% bootstrap confidence intervals generated to determine whether the estimated path coefficients were significant. All predictors in the PLS-PA were first standardized before implementing this analysis in R v4.1.1 using the “plspm” package (v4.1.1; http://cran.r-project.org/, accessed on 4 December 2022) [45,46].

## 3. Results

### 3.1. Soil Properties and Aboveground Biomass

**Figure 2:**
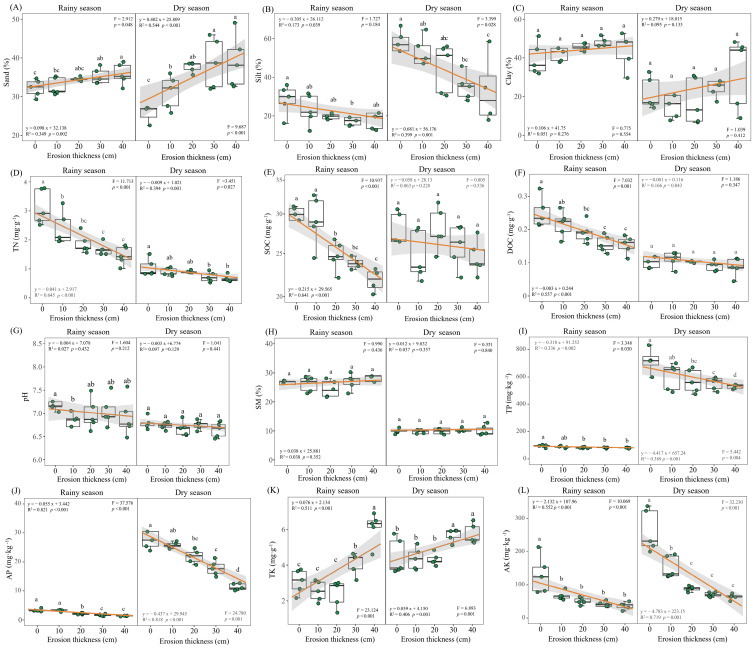
Soil physicochemical properties under differing erosion thicknesses. Different letters denote significant differences (*p* < 0.05) between the treatments according to Fisher’s least significant difference (LSD). ((**A**) the content of sand; (**B**) the content of silt; (**C**) the content of clay; (**D**) the content of TN; (**E**) the content of SOC; (**F**) the content of DOC; (**G**) the content of pH; (**H**) the content of SM; (**I**) the content of TP; (**J**) the content of AP; (**K**) the content of TK; (**L**) the content of AK). Abbreviations: TN, soil total nitrogen; SOC, soil organic carbon; DOC, dissolved organic carbon; SM, soil moisture; TP, soil total phosphorus; AP, available phosphorus; TK, soil total potassium; AK, available potassium.

**Figure 3:**
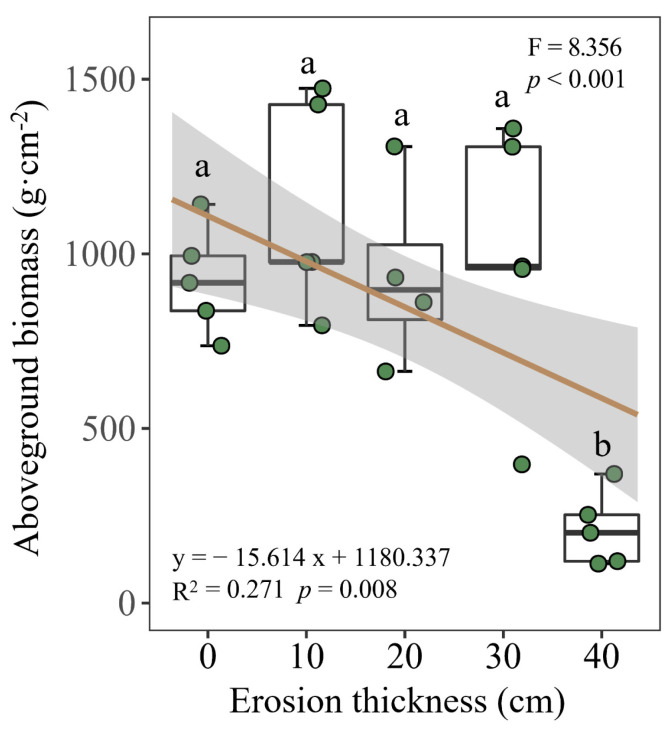
Aboveground plant biomass under differing erosion thicknesses. Different letters denote significant differences (*p* < 0.05) between treatments as determined by comparisons using Fisher’s least significant difference (LSD).

Most of the soil physicochemical properties were significantly affected (*p* < 0.05) by soil erosion thickness. Specifically, during the rainy season, with the increase in erosion thickness, the silt, SOC, DOC, TP, AP and AK contents decreased (*p* < 0.05) whereas the sand and TK contents increased (*p* < 0.05). The dry season was the same as the rainy season except for the SOC (Figure 2). Aboveground biomass decreased as the erosion thickness increased, with the biomass being much lower at the 40 cm thickness than under the other treatments (*p* < 0.05; Figure 3).

**Figure 4:**
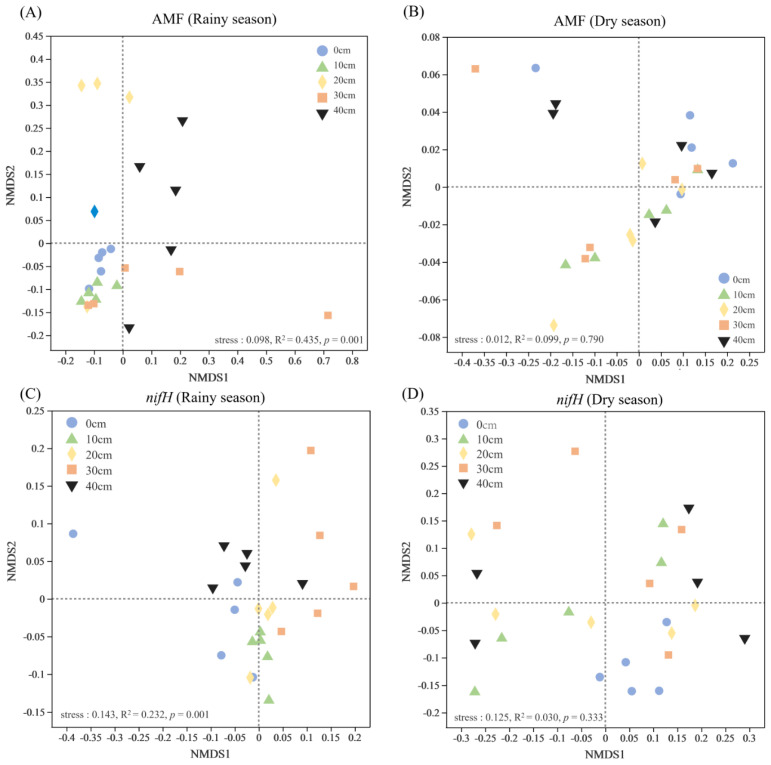
Nonmetric multidimensional scaling (NMDS) for soil microbial community compositions in the different soil erosion thickness treatments (based on the Bray–Curtis distance). The 10, 20, 30 and 40 cm inset labels denote the simulated erosion thickness applied, for which the 0 cm served as the control ((**A**) rainy season of AMF; (**B**) dry season of AMF; (**C**) rainy season of *nifH* genes; (**D**) dry season of *nifH* genes).

In all five treatments, the dominant genera of microorganisms changed greatly in the dry and rainy seasons. Specifically, except for *Diversispora*, all the dominant genera of AMF disappeared in the dry season (Figure S3). As for *nifH* genes, *Bradyrhizobium* was the dominant genus in both rainy and dry seasons, but *Azospirillum*, *Methylosinus*, *Agrobacterium*, *Rubrivivax*, *Pseudomonas* and *Anaeromyxobacter* disappeared in the dry season, and *Zohydromonas*, *Skermanella*, *Azotobacter* and *Beijerinckia* appeared (Figure S3). The NMDS analysis indicated that community composition of AMF and *nifH* genes varied significantly in response to different erosion thicknesses in the rainy season (*p* < 0.05) but not in the dry season (*p* > 0.05; Figure 4). An analysis of similarities confirmed this result (Table 1). The Shannon index of AMF decreased with greater erosion thickness but that of *nifH* genes increased (*p* > 0.05; Figure S4).

### 3.2. Soil Nitrogen Dynamics

**Figure 5:**
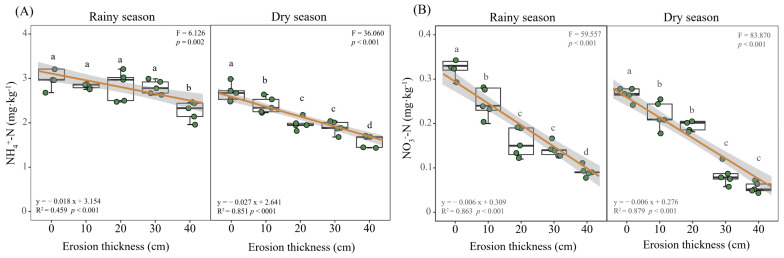
Mineral nitrogen under differing erosion thickness. Different letters denote significant differences (*p* < 0.05) between treatments as determined by comparisons with Fisher’s least significant difference (LSD). ((**A**) the content of NH_4_^+^-N; (**B**) the content of NO_3_^−^-N). Abbreviations: NH_4_^+^-N, ammonium nitrogen; NO_3_^−^-N, nitrate nitrogen.

**Figure 6:**
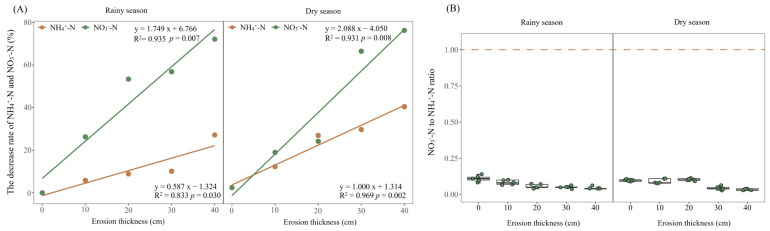
The rate of decrease (**A**) and the ratio (**B**) of mineral nitrogen across differing levels of erosion thicknesses. Abbreviations: NH_4_^+^-N, ammonium nitrogen; NO_3_^−^-N, nitrate nitrogen.

The results showed TN contents reduced with a greater soil erosion thickness, decreasing significantly at 10 cm in the rainy season, 30 cm in the dry season when compared with the 0 cm (*p* < 0.05; Figure 2D) at the early stage of vegetation self-restoration. The contents of NH_4_^+^-N and NO_3_^−^-N in soil had decreased significantly in response to increased erosion thickness (*p* < 0.05; Figure 5). NH_4_^+^-N content declined from 3.11 and 2.68 mg·kg^−1^ under no erosion (0 cm thickness) to 2.27 and 1.59 mg·kg^−1^ at a 40 cm thickness of soil erosion in the rainy and dry season, respectively, while the NO_3_^−^-N content correspondingly fell from 0.33 and 0.27 mg·kg^−1^ to 0.09 and 0.06 mg·kg^−1^. Compared with the control, at the 10, 20, 30 and 40 cm levels of soil erosion thickness, the NH_4_^+^-N was reduced by 5.84%, 8.89%, 10.14% and 27.19% in rainy season and 10.51%, 26.23%, 29.18% and 40.68% in dry season, while the NO_3_^−^-N was reduced by 26.30%, 53.40%, 56.86% and 72.17% in rainy season and 17.68%, 23.38%, 68.52% and 78.99% in dry season (Figure 6A). The NO_3_^−^-N:NH_4_^+^-N ratios were always <1, irrespective of soil erosion thickness and seasons (Figure 6B).

### 3.3. Interaction Effects of Soil Erosion, Soil and Plant Properties on Nitrogen

**Figure 7:**
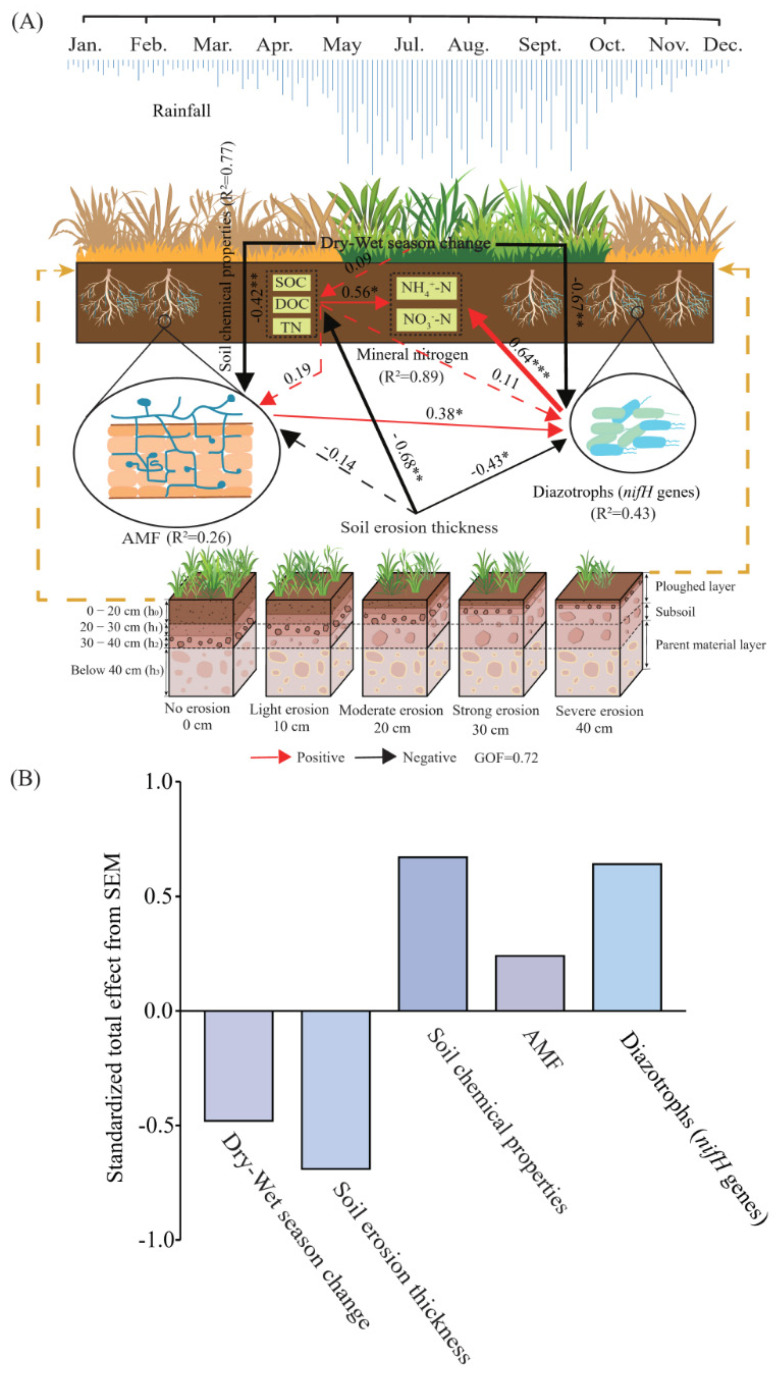
Structural equation model (**A**) showing the direct and indirect effects between mineral nitrogen and their key drivers and its (**B**) standardized total effects. Note: Continuous arrows and dashed arrows indicate significant and non-significant relationships, respectively. The significance level is denoted by * (*p* < 0.05), ** (*p* < 0.01), and *** (*p* < 0.001). Numbers adjacent to arrows indicate actual *p*-values; an arrow’s width is proportional to the size of its path coefficient. The red and black arrows indicate positive and negative relationships, respectively. Abbreviations: SOC, soil organic carbon; DOC, dissolved organic carbon; TN, soil total nitrogen; AMF, arbuscular mycorrhizal fungi; NH_4_^+^-N, ammonium nitrogen; NO_3_^−^-N, nitrate nitrogen.

SEM explained 89% of the mineral nitrogen changes in the initial stage of vegetation self-restoration on sloping farmland (Figure 7). Soil erosion thickness and dry-wet season change are the important driving factors for mineral nitrogen content, and soil erosion thickness indirectly affected mineral nitrogen through negative on total nitrogen, carbon content and *nifH* genes. Dry–wet season change effect on mineral nitrogen mediated by AMF and *nifH* genes. We also found AMF had a promotion to *nifH* genes in eroded soil, which can be expected to benefit nitrogen fixing.

## 4. Discussion

### 4.1. Effects of Soil Erosion Thickness on Soil Nitrogen Dynamics

Our results showed both soil mineral nitrogen contents decreased with an increasing erosion thickness (Figure 5). Similarly, in previous experiments simulating soil erosion, its thickness reduced soil mineral nitrogen in black soil and red soil in China [15,36]. Generally, the more severe the erosion is, the lower is the mineral nitrogen content [47]. This impact is largely ascribed to soil resource losses, which directly reduce the TN content (Figure 2D). Both NH_4_^+^-N and NO_3_^−^-N reached their minimum values under the most severe erosion level (40 cm thickness). However, Qiu et al. (2021) reported that the mineral nitrogen fell to its lowest values under moderate and severe erosion conditions in loess soils and black soils [16]. We found an increasing rate of NH_4_^+^-N or NO_3_^−^-N decline with the soil erosion thickness, but the slope of NO_3_^−^-N surpassed that of NH_4_^+^-N; this strongly suggests that NO_3_^−^-N may be more susceptible to soil erosion than soil NH_4_^+^-N (Figure 6A). Furthermore, NO_3_^−^-N can rapidly be leached from soils could be another contributing reason for its greater loss than NH_4_^+^-N. Accordingly, N limitation would possibly tend to increase with soil erosion thickness. Our N-addition experiment proved that N limitation exists at the same simulated soil erosion plots and that it is exacerbated by greater soil erosion thickness (unpublished data). The NO_3_^−^-N:NH_4_^+^-N ratios never exceeded a value of 1 in the five soil erosion thickness treatments, also indicating that N was generally scarce (Figure 6B).

### 4.2. Dominant Drivers of Soil Nitrogen

Unlike the bulk of organic nitrogen, most mineral forms of nitrogen are quite soluble in water and may be easily lost from soils through leaching and volatilization [48]. Using structural equation modeling, we find that soil erosion thickness and dry-wet season change are the key drivers of soil mineral nitrogen content at the early stage of vegetation self-restoration on sloping arable land in the dry-hot valley (Figure 7). Many researchers have reported that soil erosion and runoff can lead to losses of soil chemical properties, especially total nitrogen [5]. Here, we found the pronounced negative relationship between soil erosion thickness and soil chemical properties; of particular interest was the observed positive relationship found between soil chemical properties and mineral nitrogen. This may be because the activity of diazotrophs usually decreases at high N availability and increases at N deficiency. The *nifH* genes are often used as a molecular indicator for detecting diazotrophs because it is an important structural gene for nitrogennase [49]. Although soil erosion thickness reduces the relative abundance of *nifH* genes, *nifH* still positively impacts mineral nitrogen, implying that bolstered *nifH* abundance and activity may augment the available nitrogen in soil through nitrogen fixation, especially where soil erosion conditions are severe and soil available resources are scarce.

Our study found that the effects of dry-wet seasonal changes on mineral nitrogen are mainly mediated by AMF and *nifH* genes (Figure 7). The relative abundance of microorganisms exhibits specificity for seasonal changes. For AMF, in the rainy season, the soil moisture increases, which is benefit for vegetation growth, thus subsequent infection of AMF [50]. When it comes to the dry season, the most abundant mycelium colonization in roots and the inability of plant roots to obtain enough water and the absorption and transport of nutrients with low mobility resulted in the decrease in AMF dominant genera in the dry season. For *nifH* genes, during the transition from the rainy season to the dry season, genera that are well adapted to the environment are expected to replace those that are poorly adapted.

We also found that AMF has a catalytic effect on *nifH* genes that erode soil (Figure 7), which may be beneficial for nitrogen fixation. This result suggests AMF are conducive to promoting the functional activity of *nifH* genes during the early stage of vegetation self-restoration on sloping arable land in differing soil erosion thickness conditions in the dry-hot valley [51]. This finding also indicated that the interaction between AMF and *nifH* genes is beneficial for the accumulation of available nitrogen, which is expected to mitigate negative effects of soil erosion on mineral nitrogen. Work by Zhu et al. (2018) and Yu et al. (2021) also pointed to the interaction between AMF and *nifH* genes having a pivotal regulatory role in the soil nitrogen cycle [52,53]. The mycelium network of AMF can extend farther away to obtain more water and nutrients. Thus, even if the top layer of soil is eroded, AMF can still reach and obtain the resources they need from deeper soil [54].

We should emphasize that our simulated soil erosion thickness is not equal to a “straightforward” erosion process of topsoil. Unfortunately, it is difficult to find and apply a differing soil erosion thickness that corresponds to different soil erosion levels at a local scale with same soil type and topography or even under same climate. We also acknowledge the limitations of the simulation approach followed, which precluded us from demonstrating the actual soil erosion process and its impact on nitrogen. However, we believe our study provides a robust snapshot of the crucial role of soil erosion thickness in governing N availability, by revealing how soil erosion thickness can affect N forms in the early stage of vegetation self-restoration on typical sloping arable land in the dry-hot valley.

## 5. Conclusions

This outdoor experimental study investigated the effect of soil erosion thickness and dry-wet season change on soil mineral nitrogen early in vegetation self-restoration on sloping arable land in the dry-hot valley. Our findings provide compelling evidence that soil erosion thickness and dry-wet season change are the important factors determining the possible fates of soil mineral nitrogen. We also find that AMF had a promotion to *nifH* genes in eroded soil. We suggest that soil erosion thickness and sampling time on time scales should not be neglected when studying soil mineral nitrogen and even nitrogen cycling in the early stage of vegetation self-recovery in dry-hot valley environments. Our work highlights the importance of elucidating drivers in nitrogen-limited ecosystems, which is beneficial for devising and adopting proper measures for the effective restoration of vegetation.

## Figures and Tables

**Table 1:** Analysis of similarities between arbuscular mycorrhizal fungi (AMF) and Diazotroph (*nifH* genes) community composition in different soil erosion thicknesses (based on 999 permutations) (R^2^ is the proportion of variance explained, and *p* < 0.05 was considered significant).

	AMF				*nifH* Genes			
	Rainy Season		Dry Season		Rainy Season		Dry Season	
Vs_Group	R^2^	*p*	R^2^	*p*	R^2^	*p*	R^2^	*p*
0–10	0.400	0.021	0.098	0.373	0.249	0.012	0.251	0.024
0–20	0.437	0.034	0.148	0.300	0.164	0.139	0.153	0.284
0–30	0.252	0.095	0.118	0.282	0.336	0.004	0.305	0.060
0–40	0.404	0.007	0.068	0.447	0.207	0.014	0.163	0.245
10–20	0.512	0.039	0.009	0.911	0.152	0.183	0.041	0.784
10–30	0.216	0.217	0.131	0.375	0.374	0.008	0.110	0.465
10–40	0.489	0.009	0.052	0.640	0.359	0.009	0.059	0.678
20–30	0.293	0.031	0.129	0.379	0.167	0.192	0.083	0.536
20–40	0.273	0.044	0.066	0.561	0.131	0.348	0.052	0.752
30–40	0.204	0.080	0.042	0.750	0.246	0.038	0.043	0.812

## Data Availability

All data generated or analyzed during this study are included in this published article. The raw sequencing datasets for this study can be accessed from the NCBI Sequence Read Archive (SRA) database under accession number PRJNA1133894, PRJNA1133982, PRJNA1134283 and PRJNA1134335. The soil and plant property data of different erosion thicknesses in rainy and dry seasons can be found in Supplementary Materials (Table S1).

## Supplementary Materials

The following supplementary materials are available online at https://www.mdpi.com/article/10.3390/microorganisms12081546/s1, Table S1: Soil and plant properties data of different erosion thickness in rainy and dry seasons; Table S2: Thickness of the original soil layer remaining in the new cultivated horizon for different simulated erosion thicknesses; Table S3: Remaining volume of the original soil layer in a cultivated horizon under different erosion thicknesses; Table S4: Soil mixing process of cultivated soil composition under different erosion thicknesses; Figure S1: Total precipitation and average temperature during the experiment; Figure S2: Rarefaction curves of arbuscular mycorrhizal fungi (AMF) and Diazotrophs (*nifH* genes) in the soil of five different erosion thickness treatments ((A) rainy season of AMF; (B) dry season of AMF; (C) rainy season of *nifH* genes; (D) dry season of *nifH* genes); Figure S3: Relative abundances of the dominant genera of arbuscular mycorrhizal fungi (AMF) and Diazotrophs (*nifH* genes) in the soil of five different erosion thickness treatments ((A) rainy season of AMF; (B) dry season of AMF; (C) rainy season of *nifH* genes; (D) dry season of *nifH* genes); Figure S4: Soil microbial alpha diversity indices under differing erosion thickness. Different letters denote significant differences (*p* < 0.05) between the treatments according to Fisher’s least significant difference (LSD) ((A) rainy season of AMF; (B) dry season of AMF; (C) rainy season of *nifH* genes; (D) dry season of *nifH* genes).
