# Supplementary material for: Soil Erosion Thickness and Seasonal Variations Together Drive Soil Nitrogen Dynamics at the Early Stage of Vegetation Restoration in the Dry-Hot Valley
Source: Microorganisms. 2024 Jul 28;12(8):1546. doi: 10.3390/microorganisms12081546 (PMC11356167; doi:10.3390/microorganisms12081546)
Supplement: Supplementary file 1 [file microorganisms-12-01546-s001.zip › microorganisms-3126294-supplementary.pdf]

microorganisms

MDPI

Supplementary Materials

Table S1. Soil and plant properties data of different erosion thickness in rainy and dry seasons.

Soil erosion	Sand (%)	Slit (%)	Clay (%)	TN (mg.g^{-1})	SOC (mg.g^{-1})	DOC (mg.g^{-1})	pH	SM (%)	TP (mg.kg^{-1})	AP (mg.kg^{-1})	Tk (mg.g^{-1})	AK (mg.kg^{-1})	AB (g.cm^{3})	NH_4^+-N (mg.kg^{-1})	NO_3^--N (mg.kg^{-1})
							Rainy season								
0cm	33.83	30.00	36.18	2.6697	29.5666	0.2141	7.16	26.99	76.94	4.1529	2.1566	124.0720	/	2.9687	0.3433
0cm	29.30	26.29	44.41	3.7613	28.8770	0.2339	7.14	26.05	93.22	3.1645	3.1674	81.7700	/	3.7490	0.2857
0cm	30.77	35.98	33.25	3.7812	30.4976	0.2659	7.03	29.64	103.10	3.1335	3.6562	153.3380	/	2.9553	0.3267
0cm	32.53	16.18	51.29	2.9127	25.1876	0.3240	7.82	27.45	99.30	2.8813	3.7582	78.2920	/	2.6793	0.3816
0cm	34.71	33.35	31.94	2.5209	30.2562	0.2171	7.25	23.28	94.74	3.2465	2.6694	214.0080	/	3.2100	0.3215
10cm	35.24	19.76	45.00	1.9676	31.8250	0.2249	6.18	28.21	76.28	2.7101	1.8631	59.8380	/	2.7890	0.2767
10cm	31.36	12.23	56.41	1.9499	31.1527	0.2320	6.71	29.48	85.42	3.4005	2.5403	89.0080	/	2.8680	0.1965
10cm	34.88	21.88	43.24	3.2676	28.6874	0.2655	6.86	27.13	89.30	3.3055	2.1178	66.2080	/	3.3873	0.2406
10cm	30.59	31.59	37.82	2.7059	28.1874	0.1941	7.06	22.76	88.80	3.4770	3.1625	66.2120	/	2.8624	0.2754
10cm	31.18	30.23	38.59	2.0846	24.4980	0.1633	6.89	24.29	96.88	3.3650	3.0499	54.9940	/	2.7467	0.2333
20cm	33.95	18.47	47.58	1.7128	22.1879	0.1948	6.87	27.18	76.56	1.8280	1.9311	48.0360	/	2.5040	0.1254
20cm	34.54	20.53	44.94	1.9630	24.3084	0.2410	6.62	28.41	76.38	1.8450	2.9583	57.3080	/	2.9720	0.1934
20cm	34.36	18.12	47.53	1.5561	24.6532	0.1902	7.14	21.99	80.06	2.4080	3.0143	36.6820	/	2.4747	0.1854
20cm	35.30	21.47	43.23	2.4052	26.6703	0.1723	6.81	24.22	86.54	2.0790	1.3352	64.0200	/	3.2099	0.1455
20cm	34.59	19.82	45.59	1.6978	25.9290	0.1574	7.49	22.04	89.36	2.4300	2.8609	48.0360	/	3.0187	0.1233
30cm	33.59	17.59	48.82	1.5111	23.1533	0.1272	7.14	30.47	80.96	1.5875	4.4312	35.2460	/	2.9228	0.1447
30cm	34.53	19.23	46.23	1.5111	24.7222	0.1747	7.55	23.14	85.20	1.8635	4.4441	40.2520	/	2.6666	0.1667
30cm	38.18	15.29	46.53	1.6719	17.2917	0.1892	6.92	28.35	83.52	1.2790	3.1602	44.5240	/	2.7778	0.1444
30cm	36.59	25.29	38.12	2.0253	23.5671	0.1403	6.93	26.33	74.02	1.4785	3.7872	35.3960	/	2.6260	0.1266
30cm	33.53	14.76	51.70	1.6472	24.0670	0.1510	6.7	27.58	82.86	2.1185	4.6423	53.7020	/	2.9913	0.1333
40cm	32.12	19.65	48.24	1.7307	21.3259	0.1706	6.72	26.82	80.84	1.3025	6.4754	39.7760	/	2.4617	0.0886
40cm	39.00	21.47	39.53	1.8091	18.8778	0.1430	7.58	23.59	84.21	1.4575	4.6037	50.0080	/	2.4359	0.0874
40cm	38.12	13.47	48.41	1.4212	22.7568	0.1124	6.48	29.15	82.36	1.1960	6.3260	40.2220	/	1.9573	0.1123
40cm	34.65	12.65	52.70	1.3212	20.2742	0.1830	6.77	31.99	74.00	1.4640	6.9083	21.0680	/	2.1437	0.0888
40cm	35.24	35.17	29.59	1.0158	23.2395	0.1611	7.04	29.24	81.44	1.3835	6.1325	30.9580	/	2.3328	0.0845
							Dry season								
0cm	27.12	56.79	16.09	0.8283	27.1013	0.0834	6.79	10.20	563.02	38.8295	3.66	231.5690	1141.64	2.7210	0.2780
0cm	26.83	40.47	32.70	1.1677	31.2906	0.1182	6.99	11.01	677.28	30.4450	3.84	217.6928	994.18	2.5258	0.2635
0cm	19.30	66.53	14.18	1.5134	27.3254	0.1734	6.73	9.78	782.98	23.8910	5.35	200.0120	836.78	2.9860	0.2666
0cm	22.53	60.64	16.82	0.8653	21.4121	0.1035	6.79	8.66	673.92	25.4115	3.76	337.5360	737.02	2.4780	0.2779
0cm	17.82	53.65	28.53	0.8341	30.6355	0.0852	6.66	10.32	843.48	27.5040	5.76	323.2400	637.26	2.6710	0.2415
10cm	36.00	56.24	7.76	0.7499	33.8076	0.1052	6.82	8.50	614.78	24.5435	4.73	125.2860	1060.66	2.2255	0.2438
10cm	25.76	64.47	9.76	1.0573	22.0155	0.1288	6.62	10.15	459.62	25.3205	5.20	191.6720	803.25	2.5270	0.2080
10cm	32.24	47.12	20.65	0.8290	28.9115	0.1288	6.78	10.28	627.90	25.6455	4.36	133.0960	795.07	2.3376	0.1775
10cm	34.12	49.59	16.29	1.0044	23.2223	0.1168	6.74	10.15	480.16	26.2565	3.83	126.9580	976.44	2.6370	0.2088
10cm	27.59	26.53	45.88	0.8160	28.5322	0.0730	6.7	9.27	657.72	27.1430	3.75	186.7540	985.85	2.2480	0.2546
20cm	38.65	54.65	6.71	0.8783	27.7736	0.1033	6.53	10.08	630.50	23.2425	4.85	89.9020	1307.18	1.8150	0.1855
20cm	37.00	55.70	7.29	0.6688	30.8079	0.1320	6.7	11.16	473.06	18.9565	4.18	77.0980	932.16	1.9440	0.2015
20cm	35.59	51.29	13.12	0.9045	25.1532	0.1054	6.68	9.38	526.88	20.2155	3.95	84.4700	1624.17	2.1769	0.2440
20cm	38.53	31.88	29.59	0.8783	32.1698	0.1029	6.92	9.25	609.62	24.7387	4.43	91.4320	663.32	1.9470	0.2048
20cm	38.47	30.71	30.82	0.9698	27.7909	0.0987	6.55	10.90	445.66	22.1010	4.21	97.5800	861.27	1.9886	0.1813
30cm	38.71	35.35	25.94	0.8294	29.0839	0.0948	6.78	11.39	560.94	14.8745	5.55	66.4720	1252.87	2.0077	0.0870
30cm	32.00	45.59	22.41	0.5294	22.4637	0.0774	6.87	9.98	552.18	16.0985	5.93	77.1840	1132.00	1.6788	0.0786
30cm	45.94	36.76	17.29	0.6901	28.8942	0.0948	6.7	10.11	537.32	17.6320	5.90	61.9420	1306.51	1.8853	0.0575
30cm	32.53	30.18	37.29	0.5914	27.0323	0.0744	6.78	9.76	486.52	19.1660	5.12	77.8560	1358.67	2.0370	0.1200
30cm	44.24	27.88	27.88	0.9510	25.1187	0.1105	6.61	10.02	461.68	21.3355	5.93	69.8320	963.58	1.8677	0.0748
40cm	38.12	17.88	44.00	0.8642	22.4810	0.0443	6.76	9.46	505.76	10.5175	5.26	67.3560	462.64	1.4450	0.0433
40cm	33.24	21.23	45.53	0.6572	24.2222	0.1125	6.83	9.45	478.32	12.4385	5.42	64.3300	326.24	1.6769	0.0516
40cm	42.18	9.59	48.23	0.5721	24.0670	0.0862	6.45	12.70	573.30	10.9030	6.16	73.9940	201.32	1.6783	0.0718
40cm	49.06	34.65	16.29	0.6213	26.0841	0.1107	6.51	10.83	511.62	12.6860	6.51	62.9800	252.70	1.4328	0.0636
40cm	32.65	58.59	8.76	0.5931	28.2908	0.0834	6.68	9.13	488.10	10.1405	5.39	50.8440	119.84	1.7050	0.0487

Abbreviations: TN, total nitrogen; SOC, soil organic carbon; DOC, dissolved organic carbon; SM, soil moisture; TP, total phosphorus; AP , available phosphorus; TK, total potassium; AK, available potassium; AB, aboveground biomass; NH_4^+-N, ammonium nitrogen; NO_3^--N, nitrate nitrogen.

Table S2. Thickness of the original soil layer remaining in the new cultivated horizon for different simulated erosion thicknesses.

Original soil layers hi (cm)	Simulated erosion thickness (cm)				
	0	10	20	30	40
	Components of the original soil layer (hi) remaining in the new cultivated horizon (cm)				
0-20	20	12.12	7.34	4.45	2.69
20-30	0	7.88	4.78	2.89	1.75
30-40	0	0.00	7.88	4.78	2.89
40-100	0	0.00	0.00	7.88	12.66
Cultivated horizon (cm)	20	20	20	20	20

Table S3. Remaining volume of the original soil layer in a cultivated horizon under different erosion thicknesses.

Original soil layers h_i (cm)	Simulated erosion thickness (cm)				
	0	10	20	30	40
	The volume of original soil layer (h_i) in cultivated horizon (m^3)				
0-20	0.80	0.48	0.29	0.18	0.11
20-30	0.00	0.32	0.19	0.12	0.07
30-40	0.00	0.00	0.32	0.19	0.12
40-100	0.00	0.00	0.00	0.32	0.51
The volume of cultivated horizon (m^3)	0.80	0.80	0.80	0.80	0.80

Table S4. Soil mixing process of cultivated soil composition under different erosion thicknesses.

Original soil layers h_i (cm)	Simulated erosion thickness (cm)				
	0	10	20	30	40
	The width (b') of the original soil layer taken from the cultivation layer (m)				
0–20	2.00	1.21	0.73	0.44	0.27
20–30	0.00	1.58	0.96	0.58	0.35
30–40	0.00	0.00	1.58	0.96	0.58
40–100	0.00	0.00	0.00	1.58	2.53
The length (a) of each soil layer (m)	2	2	2	2	2

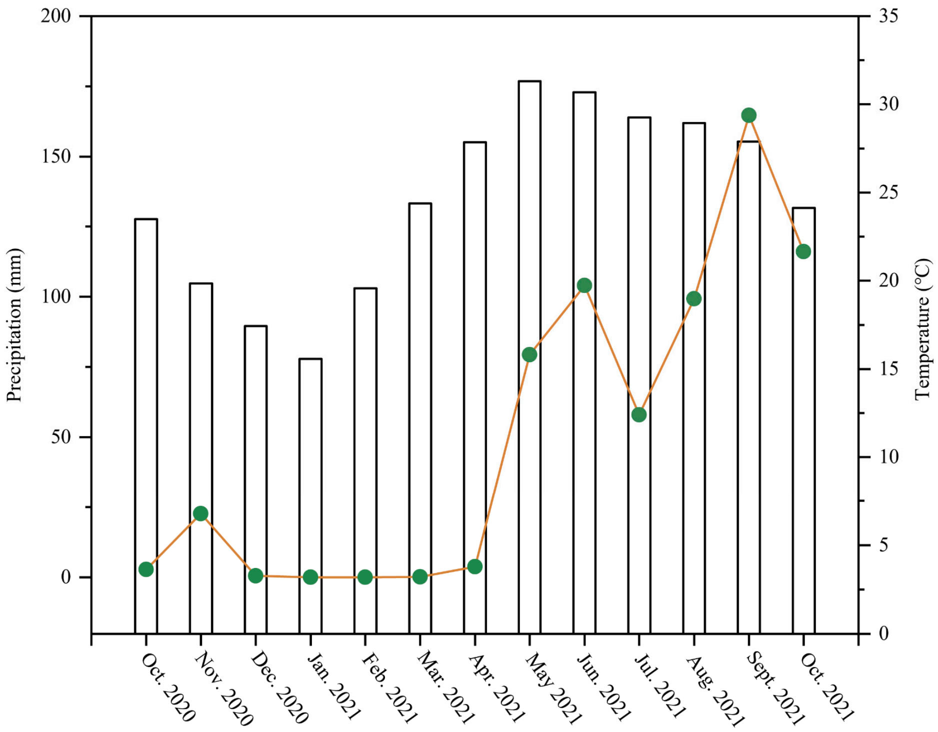

Figure S1. Total precipitation and average temperature during the experiment.

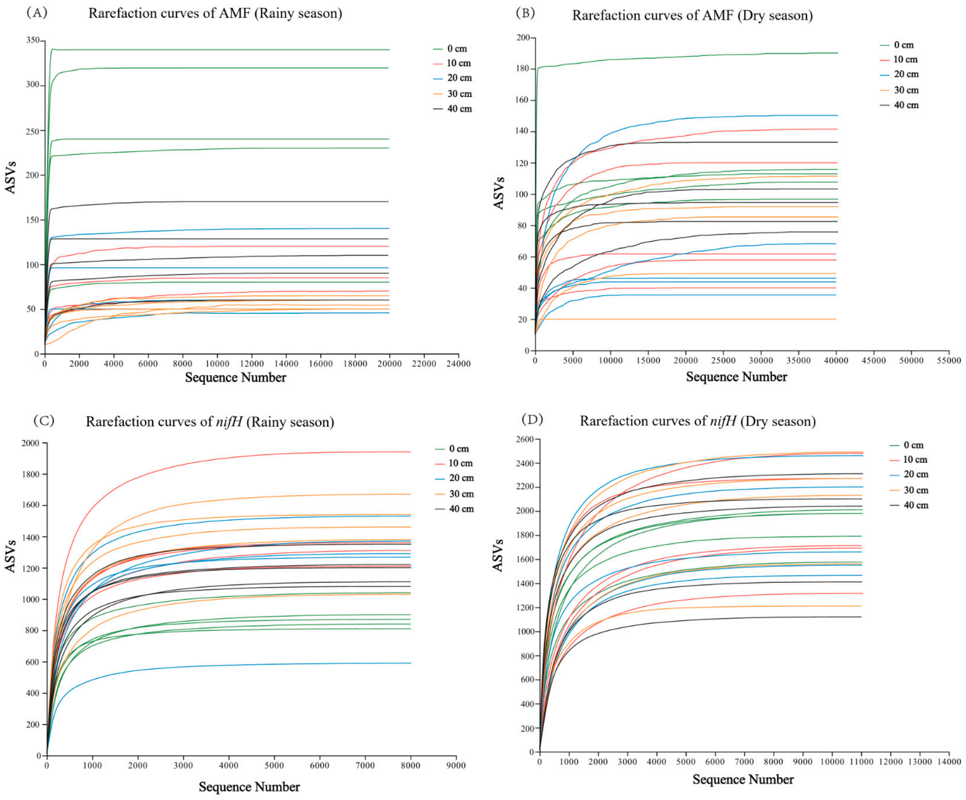

Figure S2. Rarefaction curves of arbuscular mycorrhizal fungi (AMF) and Diazotrophs (*nifH* genes) in the soil of five different erosion thickness treatments ((**A**) rainy season of AMF; (**B**) dry season of AMF; (**C**) rainy season of *nifH* genes; (**D**) dry season of *nifH* genes).

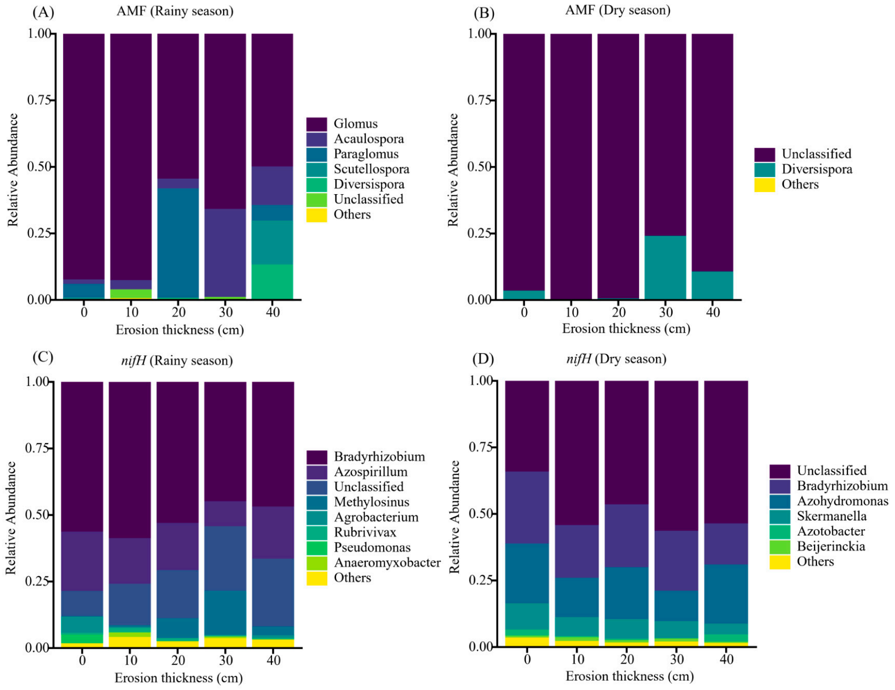

Figure S3. Relative abundances of the dominant genera of arbuscular mycorrhizal fungi (AMF) and Diazotrophs (*nifH* genes) in the soil of five different erosion thickness treatments ((**A**) rainy season of AMF; (**B**) dry season of AMF; (**C**) rainy season of *nifH* genes; (**D**) dry season of *nifH* genes).

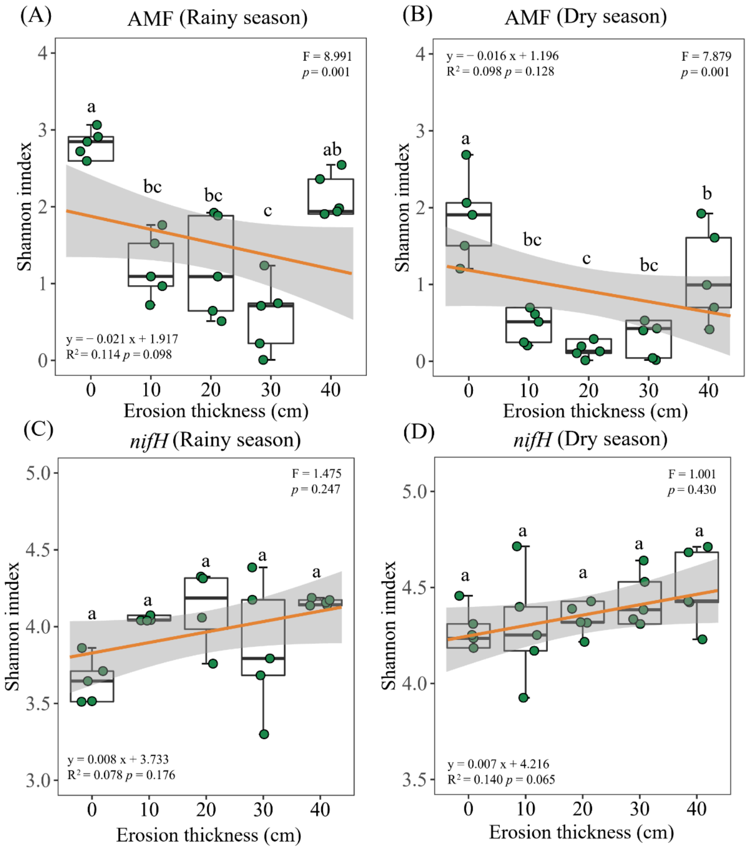

Figure S4. Soil microbial alpha diversity indices under differing erosion thickness. Different letters denote significant differences ($p < 0.05$) between the treatments according to Fisher's least significant difference (LSD) ((**A**) rainy season of AMF; (**B**) dry season of AMF; (**C**) rainy season of *nifH* genes; (**D**) dry season of *nifH* genes).
